# Transcriptome analysis of antioxidant system response in *Styrax tonkinensis* seedlings under flood-drought abrupt alternation

**DOI:** 10.1186/s12870-024-05130-4

**Published:** 2024-05-17

**Authors:** Hong Chen, Chao Han, Luomin Cui, Zemao Liu, Fangyuan Yu

**Affiliations:** Collaborative Innovation Centre of Sustainable Forestry in Southern China, College of Forestry and Grassland, College of Soil and Water Conservation, Nanjing Forestry University, Nanjing, 210037 China

**Keywords:** Flood-drought abrupt alternation, Drought stress, Antioxidant system enzymes, Reactive oxygen species

## Abstract

**Background:**

*Styrax tonkinensis* (Pierre) Craib ex Hartwich faces challenges in expanding in the south provinces of Yangtze River region due to climate extremes like flood-drought abrupt alternation (FDAA) caused by global warming. Low tolerance to waterlogging and drought restricts its growth in this area. To study its antioxidant system and molecular response related to the peroxisome pathway under FDAA, we conducted experiments on two-year-old seedlings, measuring growth indexes, reactive oxygen species content, antioxidant enzyme activity, and analyzing transcriptomes under FDAA and drought (DT) conditions.

**Results:**

The physiological results indicated a reduction in water content in roots, stems, and leaves under FDAA conditions. The most significant water loss, amounting to 15.53% was observed in the leaves. Also, ROS accumulation was predominantly observed in leaves rather than roots. Through transcriptome analysis, we assembled a total of 1,111,088 unigenes (with a total length of 1,111,628,179 bp). Generally, *SOD1* and *CAT* genes in *S. tonkinensis* seedlings were up-regulated to scavenge ROS. Conversely, the *MPV17* gene exhibited contrasting reaction with up-regulation in leaves and down-regulation in roots, leading to increased ROS accumulation in leaves. *CHS* and *F3H* were down-regulated, which did not play an essential role in scavenging ROS. Moreover, the down-regulation of *PYL*, *CPK* and *CALM* genes in leaves may not contribute to stomatal closure, thereby causing continuous water loss through transpiration. Whereas, the decreased root vigor during the waterlogging phase and up-regulated *CPK* and *CALM* in roots posed obstacles to water absorption by roots. Additionally, the DEGs related to energy metabolism, including *LHCA* and *LHCB*, were negatively regulated.

**Conclusions:**

The ROS generation triggered by *MPV17* genes was not the main reason for the eventual mortality of the plant. Instead, plant mortality may be attributed to water loss during the waterlogging phase, decreased root water uptake capacity, and continued water loss during the subsequent drought period. This study establishes a scientific foundation for comprehending the morphological, physiological, and molecular facts of *S. tonkinensis* under FDAA conditions.

**Supplementary Information:**

The online version contains supplementary material available at 10.1186/s12870-024-05130-4.

## Background

Currently, climate change is resulting in the uneven precipitation patterns worldwide and climate extremes, further posing a consequential threat to human society and ecosystem [1, 2]. The catastrophic events, including mega-floods, mega-droughts and drought-flood abrupt alternations, happen more frequently and intensively due to climate change [3, 4]. In 2021, a significant number of 223 flood incidents were recorded globally, with notable occurrences in countries such as China, India, Afghanistan, and Germany. During the same timeframe, extensive droughts prevailed across North America, Africa, and Asia, leading to extended periods of aridity. Both disasters brought reductions in crop yields and significant economic losses [5, 6]. In recent decades, China has experienced a series of severe natural disasters characterized by abrupt alternations between drought and flood, greatly influenced by the monsoon climate [7, 8]. This new type of extreme hydrological event is known as flood-drought abrupt alternation (FDAAFDAA), which means alternating occurrence of two scenarios (droughts and floods) and the state transformation is speedy [9]. FDAA events perform as two situations, which are transitioning from drought to flood and from flood to drought [10], leading to more devastating impacts on socioeconomic loss and ecological destruction than a singular occurrence of drought or flood [11].

Previous FDAA studies are mainly focused on the spatial distribution, physical mechanism, water quality and the proper water resources distribution. Ma et al. [12] pointed out that FDAA events were becoming expanding in terms of spatial distribution from frequency and intensity aspects. In order to explore the physical mechanism of FDAA, experts discover that the degree of rainfall concentration is an essential reason for FDAA happening by determining the correlation between the FDAA and precipitation indexes [13, 14]. Bi et al. [15] predicted the impacts of FDAA events on surface water quality data in Luanhe River basin for the future three decades. FDAAHuang et al. [16] reported that Guangzhou plain exhibited a prevalent and persistent arid climate throughout the entire year, coupled with a heightened vulnerability to prolonged wet conditions during the Summer-Autumn season. Besides, the frequency of FDAA events was higher during the summer months (June to August) compared to autumn or spring, with no occurrences observed during the winter season [15], indicating that the occurrence of FDAA events was seasonal.

In addition to the hydro-meteorological studies on FDAA, some researchers were addicted to investigate the influences of the compound natural disaster from agricultural perspective. Rice (*Oryza sativa* L.), being highly susceptible to the intricate interplay of water and temperature, emerges as the crop most profoundly impacted by FDAA. Consequently, rice has garnered significant research attention [2, 10, 17]. The average yield of rice under FDAA stress was reduced by 12.98% in 2016 and 29.94% in 2017, respectively [17]. Crop roots can be adversely affected by both water deficits and excess water in soils, stemming from drought and flood disasters. These conditions hinder the efficient absorption of water and essential nutrients by crop roots, consequently disrupting crop growth and reducing overall yield [18]. Both Xiong et al. [10] and Zhu et al. [2] explored the approach of rice yield recovery after FDAA via applying nitrogen. Furthermore, other significant grain and cash crops, such as cotton [18], wheat [19] and maize [7], have also been the subject of agricultural FDAA research. Nevertheless, the existing FDAA studies rarely pay attention to the impacts of FDAA events on tree species.

In our study, *S. tonkinensis*, a deciduous tree species, was utilized as the experimental objective. *S. tonkinensis* is a valuable tree species known for its economic significance, primarily due to its oil extraction potential, medicinal properties, and ornamental value [20–22]. Researchers have extensively investigated the seeds of this plant due to their remarkably high oil content, focusing on their biodiesel properties, nutritional components, and the ultrastructure of the oil bodies [23–25]. The four primary free fatty acids found were palmitic acid, stearic acid, oleic acid, and linoleic acid. Within the compounds of flavonoids, the predominate components comprised of flavans, flavonoid glycosides, and o-methylated flavonoids [26]. It is mainly distributed in the southern China, especially in Yangtze River basin [27]. Meanwhile, this versatile species is highly vulnerable to waterlogging stress, as evidenced by a 100% mortality rate of one-year-old seedlings after undergoing five days of flooding treatment [28].Affected by global climate change, the occurrence and strength of FDAA events have notably surged in the middle and lower sections of China’s Yangtze River region. Interestingly, this region witnessed a sudden shift from flooding to drought conditions around mid-July, marked by a notable change in daily precipitation patterns [29].

In the present scenario, biennial *S. tonkinensis* seedlings were subjected to FDAA to observe the morphological, physiological, and molecular responses of the species. To discern the impact of FDAA, we established control groups (CK) and subjected some seedlings to drought stress (DT), allowing for a comprehensive comparison of each treatment. The primary objective of this study was to offer a theoretical foundation for the prospective extensive cultivation of *S. tonkinensis* in the Yangtze River basin.

## Materials and methods

### Plant material and treatment

The experimental seeds were collected from Pingxiang, Jiangxi Province, China in 2020, which was mentioned in the previously published article [28]. Professor Fangyuan Yu assisted in identifying the species in Pingxiang. A voucher specimen of this material has been deposited in Chinese Field Herbarium, Shanghai, China. After two years of cultivation, the treatments were started at 9 am at the end of June 2022. The experiment was divided into three treatments, which were CK, FDAA, and DT. On the day the waterlogging treatment began, all three treatments were thoroughly watered. Subsequently, the CK seedlings received regular watering once a day, maintaining the soil relative water content between 60 and 70% (Tab.S1). The drought treatment followed the same water management as CK for the first 3 days after watering, then remained without watering for the next 7 days to simulate natural drought conditions. For the FDAA treatment, the initial flooding treatment utilized a double-pot method, with an inner permeable non-woven fabric bag and an outer impermeable flowerpot. The seedlings were flooded for 2 days, with the water level maintained at 2–5 cm above the substrate. After 2 days, the outer flowerpot was removed, and the seedlings in the non-woven fabric bags were placed on a seedbed to drain for 1 day, followed by 7 days without watering to simulate natural drought conditions. The relative soil water content after 7 days of these treatments was shown in Tab.S1.

The organic matter condition was mentioned in the previously published article [28]. Each treatment consisted of 30 seedlings. For each treatment, leaf and root samples for physiological and molecular determination were collected from 16 seedlings with destruction. The molecular samples were promptly frozen in liquid nitrogen and the physiological samples were put in ice bag. Subsequently, all of them were transferred to refrigerators at -80℃. The samples were named as CKL (leaf sample in control groups), FDAAL (leaf sample under flood-drought abrupt alternation), DTL (leaf sample under drought stress), CKR (root sample in control groups), FDAAR (root sample under flood-drought abrupt alternation), and DTR (root sample under drought stress) for transcriptome analysis. Four seedlings for each treatment were used for biomass determination. Four biological replicates were applied in the study. Besides, ten seedlings were used for observing and measuring seedling height (H) and shoot diameter (D).

### Seeding growth and seedling water content

The two measurements of H and D were taken 15 days part. For each treatment, ten random seedlings of *S. tonkinensis* were chosen to measure the H and D using a tape measure (accuracy of 0.1 cm) and a Vernier caliper (accuracy of 0.01 mm), respectively. Additionally, the height-diameter ratio was calculated.

Seedlings were first separated into their respective parts: leaves, stems, and roots. These parts were then carefully arranged in envelopes and subjected to an oven treatment. The oven temperature was set to 105 °C for 30 min initially. Subsequently, the temperature was adjusted to 70 °C to facilitate the drying process until a constant weight was achieved. Finally, precise measurements of the dry weight of each part were taken using an electronic balance with an accuracy of 0.001 g. After obtaining both fresh weight and dry weight of each organ, the water content for each organ and total water content (%) can be calculated.

### The content of H_2_O_2_, O_2_^−^ and MDA

All physiological parameters were assessed using a Lambda 365 spectrometer (PerkinElmer, Waltham, Massachusetts, USA). The determination of hydrogen peroxide (H_2_O_2_) content was carried out following the guidelines provided by the Hydrogen Peroxide assay kit (Nanjing Jiancheng Bioengineering Institute, Nanjing, China). For this assay, 0.3 g of leaf or root tissue was extracted in 2.7 mL of normal saline. After centrifugation, 0.1 mL of the resulting supernatant was combined with the provided reagent, and the optical density (OD) was measured at 405 nm. Furthermore, the soluble protein content of each sample was measured to facilitate the subsequent calculation of H_2_O_2_ content (mmol·gprot^− 1^).

The quantification of superoxide anion (O_2_^−^) content and malondialdehyde (MDA) content followed the methods described by Ma et al. [30] and Cakmak and Horst [31], respectively. For this analysis, 0.3 g of leaf or root tissue was finely ground in 8 mL of pH 7.8 phosphate buffer solution (PBS) and then subjected to centrifugation. Subsequently, 1 mL of the resulting supernatant was mixed with 0.75 mL of PBS and 0.25 mL of hydroxylamine hydrochloride, and the mixture was placed in a 25 ℃ water bath for 20 min. Following this, 2 mL each of 17 mmol·L^− 1^ 4-aminobenzenesulfonic acid and 7 mmol·L^− 1^ 1-Naphthylamine naphthylamine were added to the solution. The sample was then incubated in a 30 ℃ water bath for 30 min, and OD at 530 nm was recorded.

To determine the MDA content, 0.3 g of leaf or root tissue was extracted using 5 mL of 10% trichloroacetic acid (TCA) and then subjected to centrifugation. Next, 2 mL of the resulting supernatant was mixed with 4 mL of 0.6% thiobarbituric acid and boiled for 20 min. After the solution cooled to room temperature, OD at 450 nm, 532 nm, and 600 nm were recorded. These values were then utilized in the calculation to determine the MDA content.

### Histochemical detection of H_2_O_2_ and O_2_^-^

In the study conducted by Kaur et al. [32], the localization of H_2_O_2_ and O_2_^−^ in leaf samples was investigated using histochemical detection methods. To visualize the location of H_2_O_2_, the leaves were immersed in a solution containing 3,3’-diaminobenzidine (DAB) while exposed to light for 12 h at room temperature. To capture the location of O_2_^−^, a solution of 6 mM nitrozolium blue tetrachloride (NBT) mixed in sodium citrate buffer was utilized. Subsequently, the treated leaves were incubated at room temperature for a duration of 12 h. Eventually, both leaf samples for histochemical detection of H_2_O_2_ and O_2_^−^ were transferred to ethanol and boiled at 100℃ to eliminate chlorophyll interference. To prevent dehydration, the treated leaves were then placed in a 20% glycerol solution.

### Enzyme activities

To assess superoxide dismutase (SOD; EC 1.15.1.1) activity, the supernatant used for analysis was obtained by grinding and centrifuging 0.3 g of leaf or root tissue in 8 mL PBS at pH 7.8 Then, 0.05 mL of the supernatant was subjected to a reaction with specific chemical reagents following the NBT-illumination method [33]. The absorbance at 560 nm (OD560 nm) was recorded, and SOD activity was expressed as U·g^− 1^FW.

In another extraction process, enzyme extract was obtained from the sample using a pH 7.0 buffer and 8 mL of PBS to measure catalase (CAT; EC 1.11.3.6) activity. The 0.02 mL of leaf supernatant and 0.1 mL of root supernatant was used for H_2_O_2_ degradation, which was modified according to Ma et al. [30].

### RNA extraction and cDNA library construction

RNA extraction from leaf and root samples was carried out using the Ambion Plant RNA Kit, adhering to the protocol recommended by the manufacturer (Thermo Fisher Scientific, MA, Waltham, USA). For the evaluation of RNA integrity, the Agilent 2100 Bioanalyzer manufactured by Agilent Technologies in Santa Clara, CA, USA, was employed for the analysis. Libraries were generated using the TruSeq Stranded mRNA LT Sample Prep Kit from Illumina, based in San Diego, CA, USA, in accordance with the manufacturer’s instructions.

### Quality control, *de novo* assembly and functional annotation

Transcriptome sequencing and analysis were conducted by OE Biotech Co., Ltd. in Shanghai, China, utilizing the Illumina HiSeq 4000 Sequencing platform. The raw reads underwent processing with Trimmomatic [34] to eliminate reads containing poly-N and low-quality sequences, aiming to obtain clean reads. Trinity was used to assisting in de novo assembly of clean reads in the paired-end method [35], generating expressed sequence tag clusters (contigs) and transcripts. By comparing the length and similarity of transcript, the longest one for each cluster was chosen for subsequent analysis.

To annotate unigenes function, they were aligned with databases such as the Swiss-Prot protein (SwissProt), clusters of orthologous groups (KOG), and evolutionary genealogy of genes: non-supervised orthologous groups (eggNOG) using basic local alignment search tool (BLAST) [36] with a threshold E-value of 10^− 5^. Functional annotations were assigned to the unigenes based on the proteins showing the highest sequence similarity. Furthermore, gene ontology (GO) classification was performed based on the SwissProt annotation, establishing the mapping relationship between SwissProt and GO terms. Additionally, the unigenes were mapped to the Kyoto encyclopedia of genes and genomes (KEGG) database [37] to annotate their potential metabolic pathways.

### Differential expression analysis of unigenes and qRT-PCR analysis

The DESeq2 method was employed to normalize the gene count data for each sample, and the expression level was estimated via the base mean value, represented as fragments per kilobase per million mapped reads (FPKM). Additionally, the fold change (difference multiple) was calculated, and the significance of the differences was assessed using the negative binomial (NB) distribution test. To identify the differentially expressed genes (DEGs), the results from the difference multiple and significance tests were used for screening, following the approach described by Love et al. [38]. DEGs were deemed statistically significant if they had a p-value less than 0.05 and | log2FC | greater than 1, as the method proposed by Anders and Huber [39].

For validation of the RNA-seq results, a subset of transcripts (*MPV17, PMP34, PEX3, PEX14, SOD1, SOD2, CAT, POD*) associated with the antioxidant system were selected and verified. The primers for each of the DEGs were provided in Tab.S3. The quantitative real-time PCR (qRT-PCR) reactions were performed on a StepOne Real-Time PCR System utilizing SYBR Green Dye from Applied Biosystems (Foster City, USA) and Takara (Dalian, China). The 2^^−ΔΔCt^ method with 18 S ribosomal RNA serving as an internal control was applied to determine the relative gene expression.

### Statistical analysis

The data analysis comprised initial basic descriptive analysis, followed by an analysis of variance (ANOVA) to assess the differences between groups. Subsequently, Duncan and Pearson R correlation tests were conducted using SPSS version 23.0 for Windows (SPSS Science, Chicago, IL, USA). In evaluating significance between treatments, p-values less than 0.05 were considered indicative of statistically significant differences.

## Results

### Impacts of FDAA on the growth and water content of *S. tonkinensis*

As shown in Fig.S1, the stems and twigs of *S. tonkinensis* seedlings became curved and the leaves dropped under both FDAA and DT stress. The experimental period was during the rapid growth period of *S. tonkinensis* seedlings. *S. tonkinensis* seedlings under normal water management (CK) grew rapidly, with an increment of 13.83% in H and 5.41% in D. However, both FDAA and DT treatments inhibited the growth of *S. tonkinensis* seedlings, as evidenced by reduced H and D. The growth of H and D in the FDAA treatment was inhibited by 2.2 cm (3.48%) and 0.31 mm (4.56%), respectively. Compared to FDAA, DT stress caused even greater growth reductions in H and D (Table 1). Regarding biomass, both FDAA and DT treatments significantly inhibited fresh weight growth, including root, stem, leaf, and total biomass. As for dry weight, no noteworthy distinctions were noted among the treatments (Tab.S2).

*S. tonkinensis* seedlings under normal water management maintained a total water content of 74.43%, with the leaf exhibiting the highest water content at 76.53% among the three organs. Both FDAA and DT treatments resulted in decreased water content in the root, stem, leaf, and overall. Compared to the CK, the DT treatment had the most significant impact on total (43.72%) and root (46.68%) water content, declining by 41.26% and 36.56%, respectively. Under the FDAA treatment, the leaf experienced the most severe water loss at 15.53%, representing an almost 80% decrease compared to CK (Table 2).

**Table 1:** The variation of seedlings height and shoot diameter of *S. tonkinensis* in response to FDAA and DT between pre-treatment and post-treatment. Values are mean ± SD, *n* = 4. Different lowercase letters within each treatment indicate significant differences (*P* < 0.05)

Treatment	Pre-treatment			Post-treatment		
	H (cm)	D (mm)	Height-diameter ratio (%)	H (cm)	D (mm)	Height-diameter ratio (%)
CK	59.3 ± 2.30a	6.65 ± 0.18a	89.05 ± 2.11a	67.5 ± 2.29a	7.01 ± 0.19a	96.37 ± 1.98a
FDAA	63.2 ± 2.50a	6.80 ± 0.33a	94.03 ± 3.86a	61.0 ± 2.35ab	6.49 ± 0.30a	95.02 ± 3.97a
DT	62.9 ± 3.04a	6.80 ± 0.37a	93.45 ± 3.59a	59.6 ± 2.40b	5.73 ± 0.26b	105.06 ± 24.41a

**Table 2:** The root, stem, leaf and overall water content of *S. tonkinensis* in response to FDAA and DT. Values are mean ± SD, *n* = 4. Different lowercase letters within each treatment indicate significant differences (*P* < 0.05)

Treatment	Seedling water content (%)			
	Overall	Root	Stem	Leaf
CK	74.43 ± 0.24a	73.58 ± 0.58a	72.84 ± 0.93a	76.53 ± 0.15a
FDAA	53.83 ± 1.17b	58.51 ± 3.47b	59.16 ± 0.41b	15.53 ± 1.20b
DT	43.72 ± 3.98c	46.68 ± 4.44c	50.84 ± 5.37b	16.45 ± 0.86b

### Impacts of FDAA on ROS and lipid peroxidation of *S. tonkinensis*

**Fig. 1:**
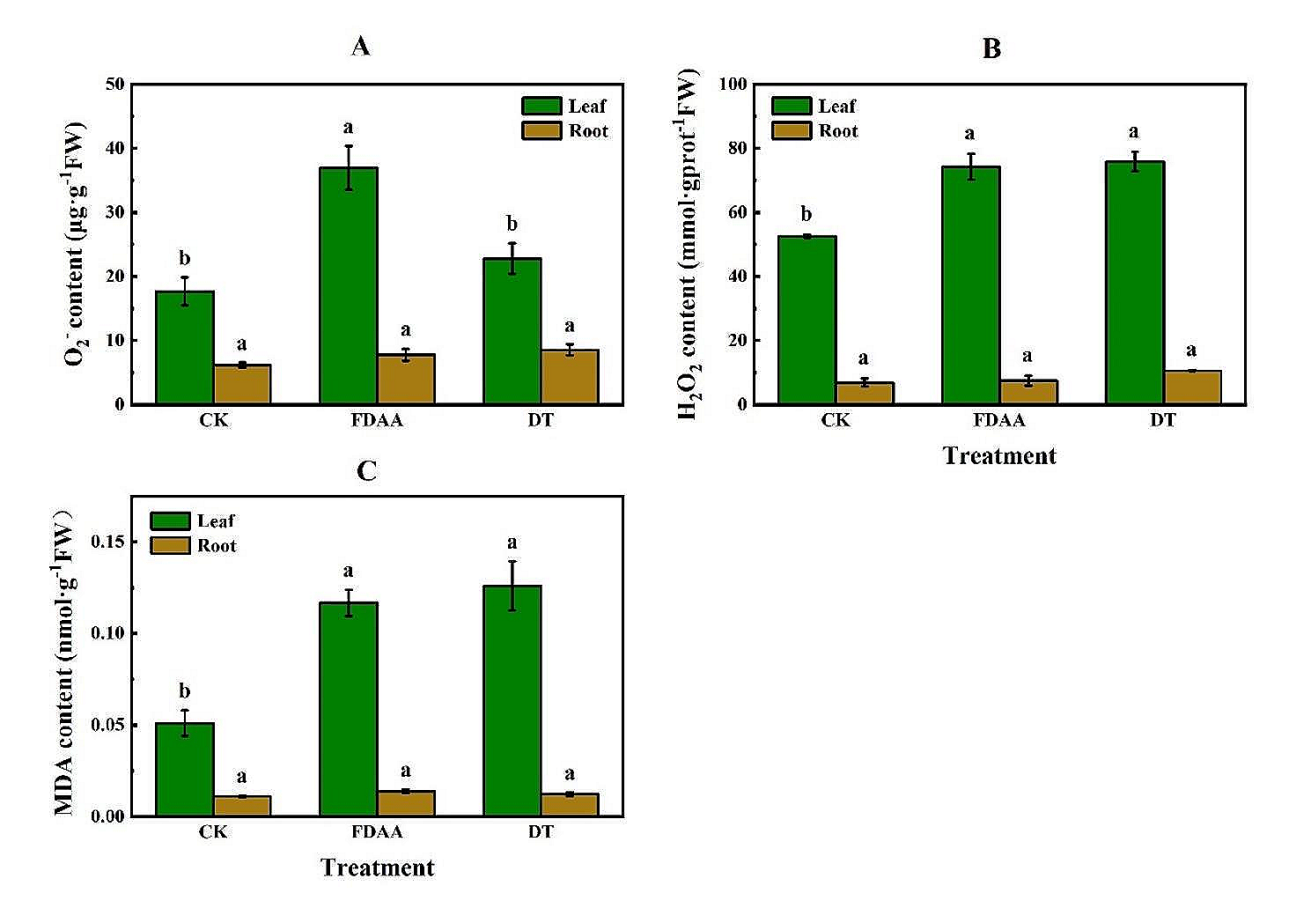
O_2_^−^ content (A), H_2_O_2_ content (B) and MDA content (C) in roots (brown) and leaves (green) of *S. tonkinensis* in response to FDAA and DT. Values are mean ± SD, *n* = 4. Different lowercase letters within each treatment indicate significant differences (*P* < 0.05). FW, estimated fresh weight

Under FDAA and DT stress, the O_2_^−^ content in both leaves and roots was elevated compared to the CK (Fig. 1A). Histochemical detection of O_2_^−^ (Fig. 1A) further confirmed that the leaves in the FDAA group were most severely attacked by O_2_^−^ with a concentration of 36.97 µg·g^− 1^FW, which was over twice as much as that in the CK group. The blue dyeing on leaves of the DT group also indicated an increase in O_2_^−^ content level (22.82 µg·g^− 1^FW). Moreover, the H_2_O_2_ content in leaves increased significantly under both FDAA and DT stress, with separate increments of 21.76 mmol·gprot^− 1^FW and 23.36 mmol·gprot^− 1^FW (Fig. 1B), as also evidenced by the brown dots on leaves in Fig.S1. In general, both O_2_^−^ and H_2_O_2_ contents in roots increased slightly under FDAA and DT stress, but without significant differences when compared to the CK. Furthermore, compared to the CK, the leaves in both FDAA and DT groups experienced severe lipid peroxidation, indicated by the dramatic elevation in MDA content. However, the variation in MDA content in roots among treatments was not significant (Fig. 1C).

### Impacts of FDAA on two antioxidant enzymes of *S. tonkinensis*

**Fig. 2:**
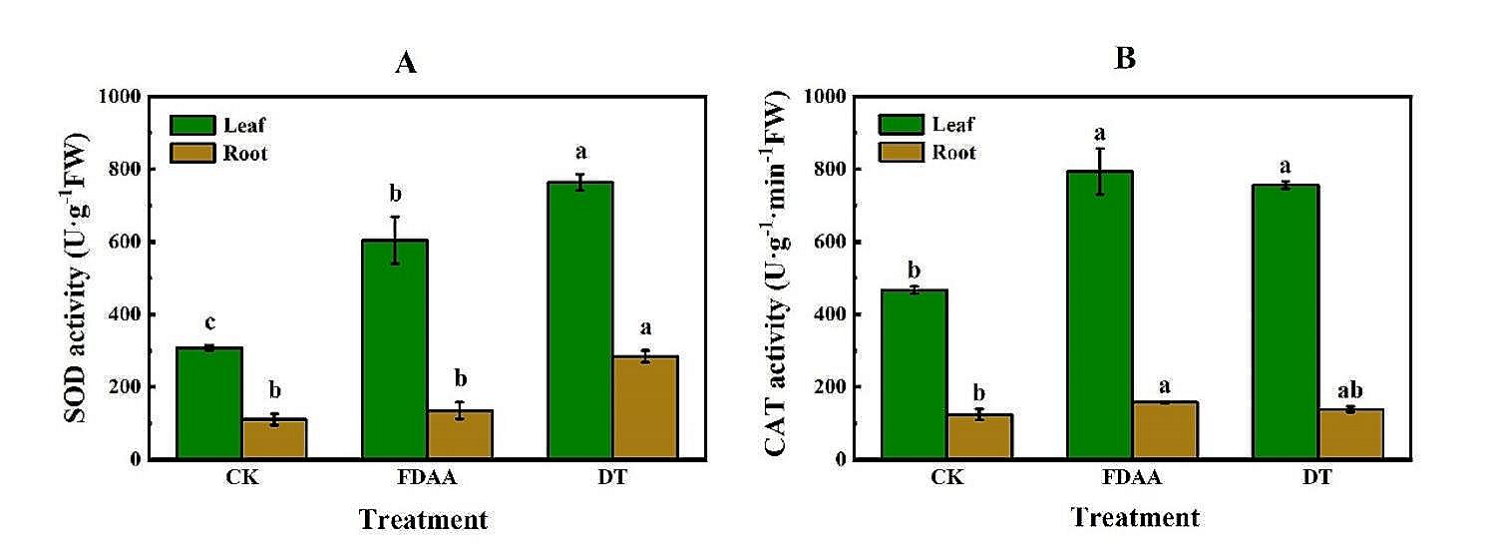
The SOD activity (A) and CAT activity (B) in roots (brown) and leaves (green) of *S. tonkinensis* in response to FDAA and DT. Values are mean ± SD, *n* = 4. Different lowercase letters within each treatment indicate significant differences (*P* < 0.05). FW, estimated fresh weight

Generally, DT led to the highest SOD activity in leaves (763.76 U·g^− 1^FW) and roots (284.58 U·g^− 1^FW) compared to other two treatments. The SOD activity in leaves and roots of FDAA was 20.85% and 52.49% lower than that of DT, respectively. Compared to CK, FDAA not only improved the SOD activity in leaves but also in roots (Fig. 2A). Regarding CAT activity, FDAA stress contributed to the maximal CAT activity in both leaves (794.44 U·g^− 1^·min^− 1^FW) and roots (158.22 U·g^− 1^·min^− 1^FW). The CAT activity of DT was slightly lower than that of FDAA in two organs, but still higher than that of CK (Fig. 2B).

### Quality control, *de novo* assembly, and total gene expression

After the completion of transcriptome sequencing for 24 samples, a cumulative total of 156.98 G of high-quality data were acquired. The individual sample datasets exhibited effective data sizes spanning from 5.97 to 6.81 G, with Q30 bases accounting for a range of 93.07–93.66%. Moreover, the collective average GC content was measured at 46.99%, as detailed in Tab.S4. The assembly process resulted in the creation of 1,111,088 distinct unigenes, with an overall length of 1,111,628,179 bp and an average length of 1004.86 bp, as outlined in Tab.S5. The dataset consisted of sequences spanning lengths between 301 and 400, with the highest count of sequences (30,690) falling within this range. Additionally, there were 14,355 sequences with lengths exceeding 2,000, which secured the second-highest count (Fig.S2B). Validation of the FPKM values was conducted and is visually depicted in Fig.S2A. For the determination of FPKM values across 24 samples derived from CK, DT, and FDAA groups, the DESeq2 method was employed. The distribution of FPKM values among these 24 samples is illustrated in Fig.S3.

### Functional annotation and classification

The BLAST program against five publicly accessible protein databases was applied to elucidate and characterize potential functions, employing a threshold E-value of 10^− 5^. The results revealed substantial matches with known proteins in the SwissProt, KEGG, KOG, eggNOG, and GO databases, yielding a total of 50,402 (45.37%), 18,284 (16.46%), 39,673 (35.71%), 64,050 (57.66%), and 44,622 (40.17%) annotated unigenes, respectively.

A comprehensive total of 44,622 assembled unigenes were systematically categorized across three principal functional domains in GO framework. These domains encompassed biological processes (37,140 unigenes, 83.23%), cellular components (40,281 unigenes, 82.35%), and molecular functions (38,829 unigenes, 87.02%) as depicted in Fig.S4A. The biological process category was further subdivided into 23 distinctive sub-categories. Among these, the two most prominently represented sub-categories were “cellular process” and “metabolic process,” housing a substantial 30,585 unigenes (69.65%) and 25,362 unigenes (68.29%) respectively. Within the cellular component category, allocation to 14 sub-categories transpired. The preponderance of unigenes were affiliated with the “cell” category (37,026 unigenes, 91.92%), closely followed by the “cell part” category (36,948 unigenes, 91.73%). Meanwhile, the molecular function domain exhibited a distribution across 16 sub-categories. Notably, the two most prevailing sub-categories were “binding” (25,937 unigenes, 66.80%) and “catalytic activity” (22,935 unigenes, 59.07%).

A total of 18,284 unigenes were categorized into five KEGG categories, 29 sub-categories, and 136 KEGG pathways (Fig.S4B). In “environmental information processing” category, the “signal transduction” pathway (743 unigenes, 4.06%) may be related to *S. tonkinensis* responding to FDAA and DT stress. In “metabolism” category, a total of 3,544 unigenes (19.38%) were assigned in the “carbohydrate metabolism” pathway, followed by “amino acid metabolism” (1,951 unigenes, 10.67%) and “energy metabolism” (1,779 unigens, 9.73%) pathways.

A comprehensive count of 39,673 unigenes underwent allocation across 25 KOG classifications, with the greatest representation observed in the “general function prediction only” category (7,509 unigenes, 18.93%). This was pursued by a notable presence in the “posttranslational modification, protein turnover, chaperones” category (4,871 unigenes, 12.28%), and subsequently in the “signal transduction mechanisms” category (3,728 unigenes, 9.40%) as visually represented in Fig.S4C. Moreover, “signal transduction mechanisms” might be connected to the response of *S. tonkinensis* to FDAA and DT stress.

### Analysis of gene expression

**Fig. 3:**
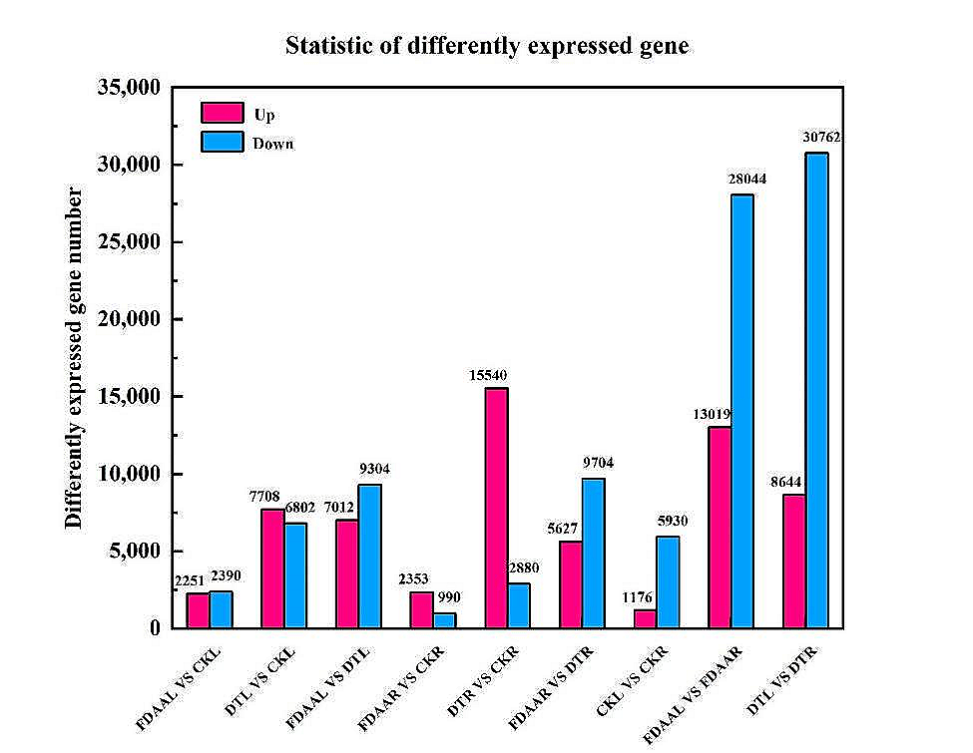
Number and distribution of up-regulated and down-regulated differently expressed genes in roots and leaves of *S. tonkinensis* seedlings between different treatments (CK, FDAA and DT)

To explore the expression patterns of differently expressed genes (DEGs) and specific pathways under FDAA and DT stress, the transcriptome profiles from treatments were compared. Compared to CKL, FDAAL possessed 2,251 up-regulated DEGs and 2,390 down-regulated DEGs. A total of 7,012 DEGs and 9,304 DEGs were positively regulated and negatively regulated in FDAAL VS DTL group, respectively. DTR had over 15,000 up-regulated DEGs when compared to CKR. Furthermore, the leaves of *S. tonkinensis* exhibited higher number of down-regulated DEGs compared to the roots, regardless of the treatment conditions (Fig. 3).

**Fig. 4:**
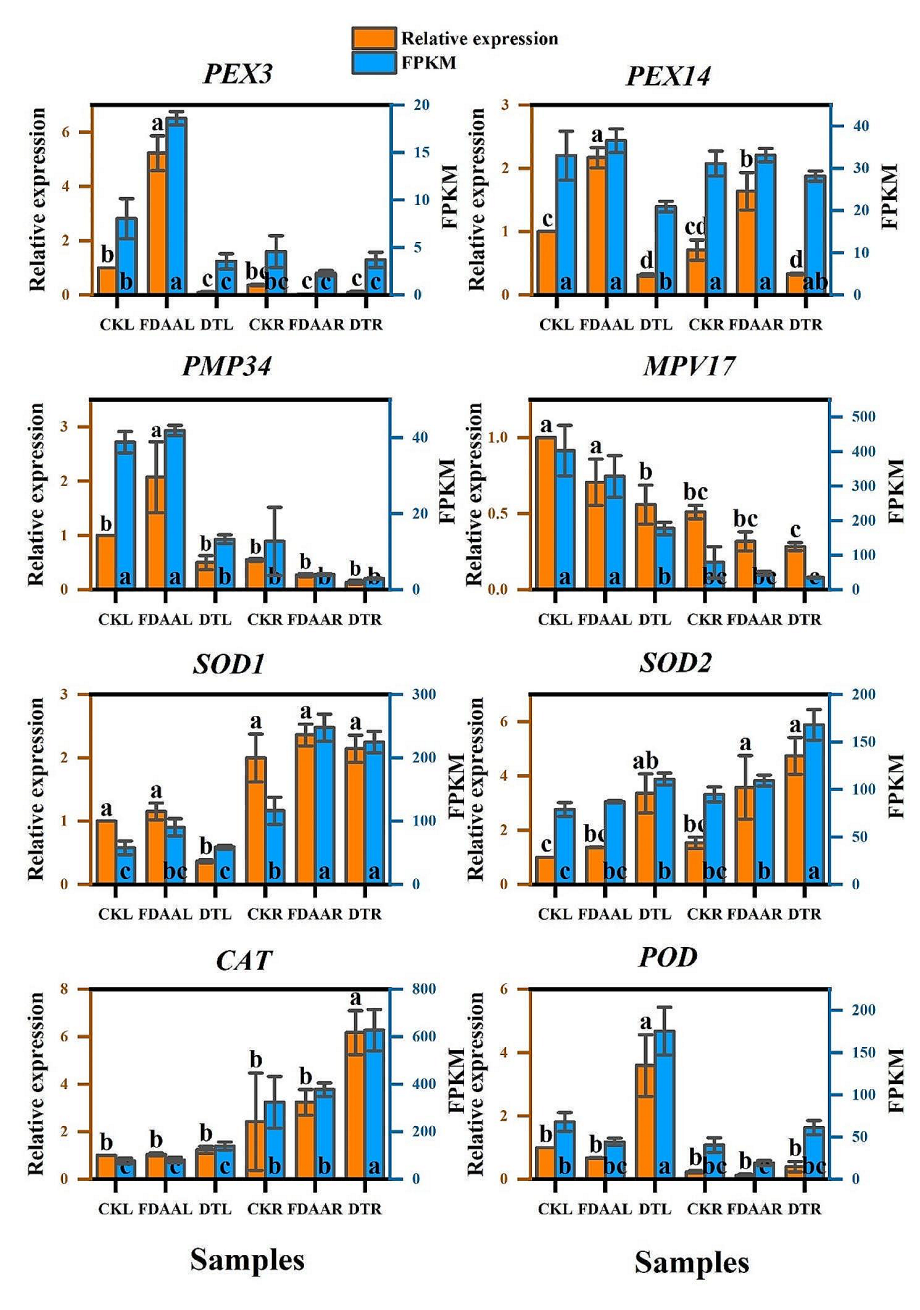
The relative expression (orange bars) from qRT-PCR and the FPKM (blue bars) from transcriptome profile of *PEX3, PMP34, MPV17, PEX14, SOD1, SOD2, CAT, POD* in both leaves and roots of *S. tonkinensis* in response to FDAA and DT. Values are mean ± SD, *n* = 4. Different lowercase letters among samples indicate significant differences (t-test; *P* < 0.05). Abbreviations: *PEX3*, peroxin-3; *PMP34*, peroxisomal adenine nucleotide transporter; *MPV17*, protein Mpv17; *PEX14*, peroxin-14; *SOD1*, superoxide dismutase, Cu/Zn family [EC:1.15.1.1]; *SOD2*, superoxide dismutase, Fe/Mn family [EC:1.15.1.1]; *CAT*, catalase [EC:1.11.1.6]; *POD*, peroxidase [EC:1.11.1.7]

The DEGs related to ROS and antioxidant system were analyzed, and their expression pattern was verified by qRT-PCR in Fig. 4. In addition, Pearson correlation ratio between the relative expression of eight genes from qRT-PCR and the corresponding FPKM from transcriptome was calculated. The result indicated a highly and positively obvious correlation (0.534, *p* < 0.01) to verify the accuracy of transcriptome data. Under FDAA condition, peroxin-3 (*PEX3*) in leaves of *S. tonkinensis* exhibited the highest expression level (18.60), and showed a significant difference compared to other samples. Compared to CKL, *PEX3* in DTL was apparently down-regulated. No significant differences were observed among the root samples. The FPKM of *PMP34* in FDAAL (41.87) went up slightly, while it in DTL (13.28) was reduced massively when compared to CKL (38.83). In the case of CKR, both FDAAR and DTR declined the *PMP34* FPKM as an adaptation to abiotic stresses. Both leaves and roots of *S. tonkinensis* improved *SOD1* and *SOD2* expression level to scavenge ROS. FDAA stress was more inclined to trigger *SOD1* expression while DT stress tended to induce *SOD2* expression. For leaves, the variation of *CAT* expression was unobvious. However, DT stress induced the highest FPKM (627.11) of *CAT* in the roots. In addition, DT stress triggered the expression of *POD* no matter in leaves or roots of *S. tonkinensis*. On the contrary, FDAAL and FDAAR showed lower FPKM values of *POD* than that of CKL and CKR, respectively.

**Fig. 5:**
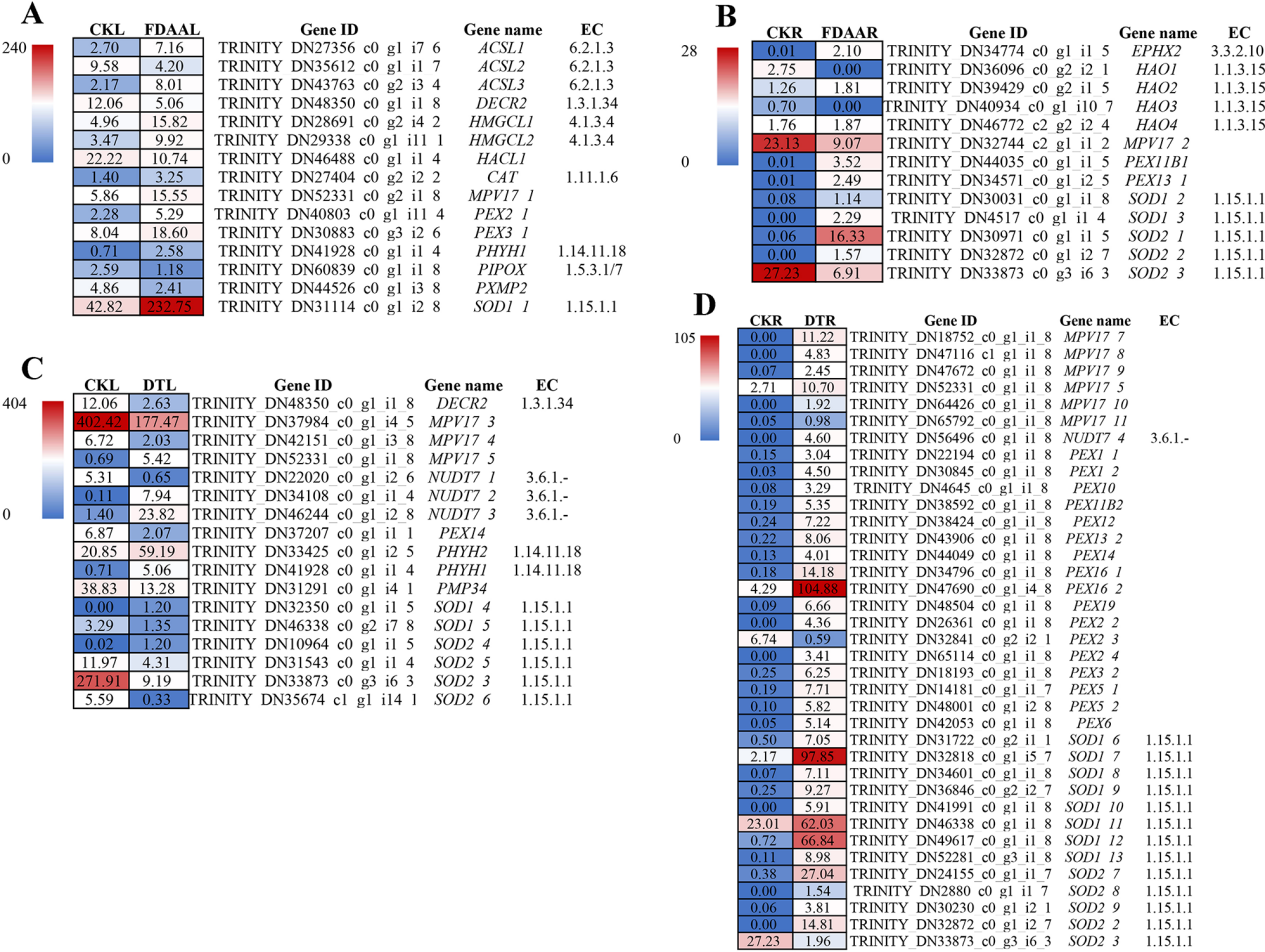
The gene expression level of key DEGs comparison in FDAAL VS CKL (A), FDAAR VS CKR (B), DTL VS CKL (C), and DTR VS CKR (D) in the peroxisome pathway of *S. tonkinensis* seedlings under FDAA and DT stress. The numbers in blocks represent the average FPKM values of same gene of samples in each treatment after deleting the data with poor repeatability. Red blocks represent high expression of DEGs, and blue blocks represent a low expression of DEGs. These DEGs need to meet the threshold that *p* < 0.05 and |log2FC| > 1

In general, drought stress contributed to the most varied expression of DEGs in the peroxisome pathway in the roots, including 40 types of DEGs (Tab.S7). In the case of Protein Mpv17 (*MPV17*), which encodes a peroxisomal protein to produce ROS, was up-regulated with one unigene in leaves, while down-regulated with one unigene in roots under FDAA. Under DT stress, three DEGs of *MPV17* were negatively regulated on the average in leave. On the contrary, six DEGs of *MPV17* were all positively regulated in roots (Fig. 5).

**Fig. 6:**
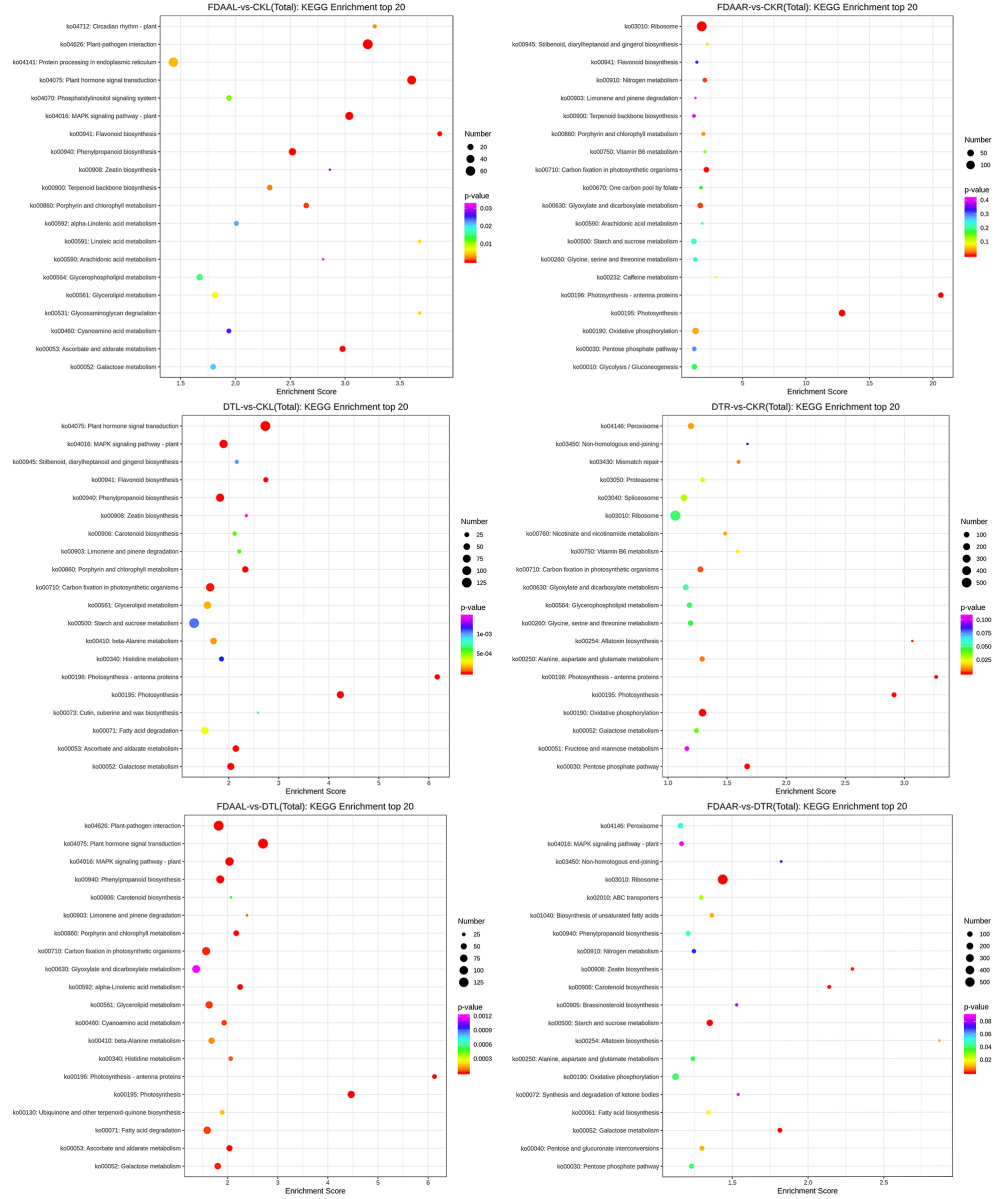
KEGG enrichment analysis of top 20 pathways of *S. tonkinensis* DEGs for six groups (FDAAL VS CKL, FDAAR VS CKR, DTL VS CKL, DTR VS CKR, FDAAL VS DTL, and FDAAR VS DTR) in response to FDAA and DT

**Fig. 7:**
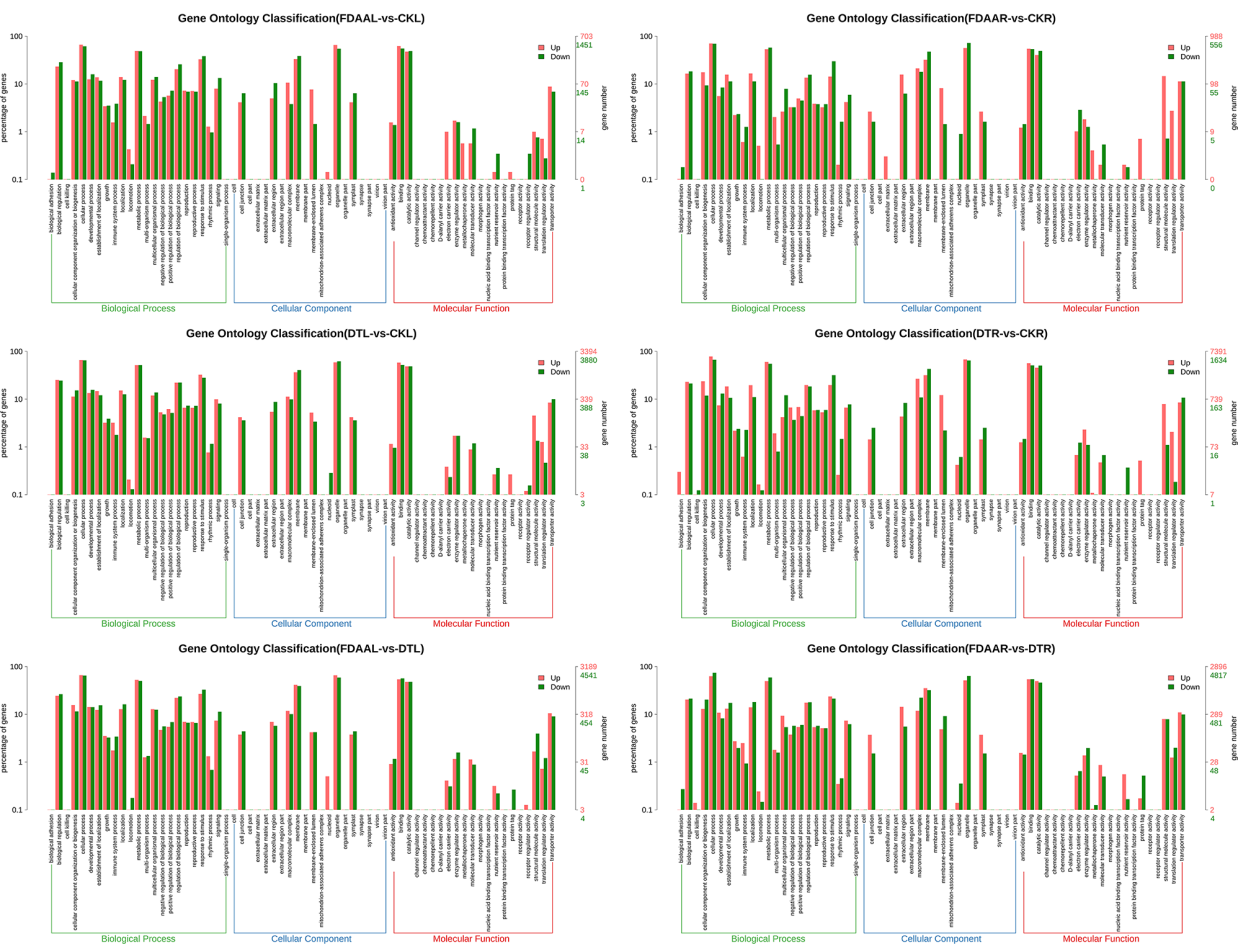
GO enrichment analysis of up/down differentially expressed genes (DEGs) of *S. tonkinensis* unigenes for six groups (FDAAL VS CKL, FDAAR VS CKR, DTL VS CKL, DTR VS CKR, FDAAL VS DTL, and FDAAR VS DTR) in response to FDAA and DT

Through KEGG enrichment analysis, the “flavonoid biosynthesis” pathway was most enriched (3.86 enrichment score) with hitting 12 DEGs in the leaves of *S. tonkinensis* under FDAA stress. Furthermore, the “plant hormone signal transduction” pathway and “MAPK signaling pathway – plant” pathway ranked 4th and 7th in terms of enrichment scores, respectively, and they were associated with abiotic adaption. For roots, FDAA stress induced the “photosynthesis - antenna proteins” pathway most enriched (20.63 enrichment score) with 28 DEGs, followed by the “photosynthesis” pathway with 12.84 enrichment score via hitting 50 DEGs. In top 20 pathways in FDAAR VS CKR, most pathways were related to energy metabolism. Concerning DT stress, the leaves of *S. tonkinensis* in DTL VS CKL had the same most enrichment pathways in FDAAR VS CKR. In DTR VS CKR, the “photosynthesis - antenna proteins”, “aflatoxin biosynthesis” and “photosynthesis” pathways ranked top3. The “peroxisome” pathway acquired 1.16 enrichment score with 106 DEGs when comparing FDAAR and DTR samples. In general, FDAA and DT stress obviously affected the enrichment of the “photosynthesis - antenna proteins” and “photosynthesis” pathways to different extent. Furthermore, the pathways related to environmental adaption were also enriched, such as the “Plant hormone signal transduction” and “MAPK signaling pathway – plant” pathways (Fig. 6). As displayed in Fig. 7, no matter in FDAA stress or DT stress, the DEGs in the “response to stimulus” (biological process) and “antioxidant activity” (molecular function) pathways were triggered obviously.

**Fig. 8:**
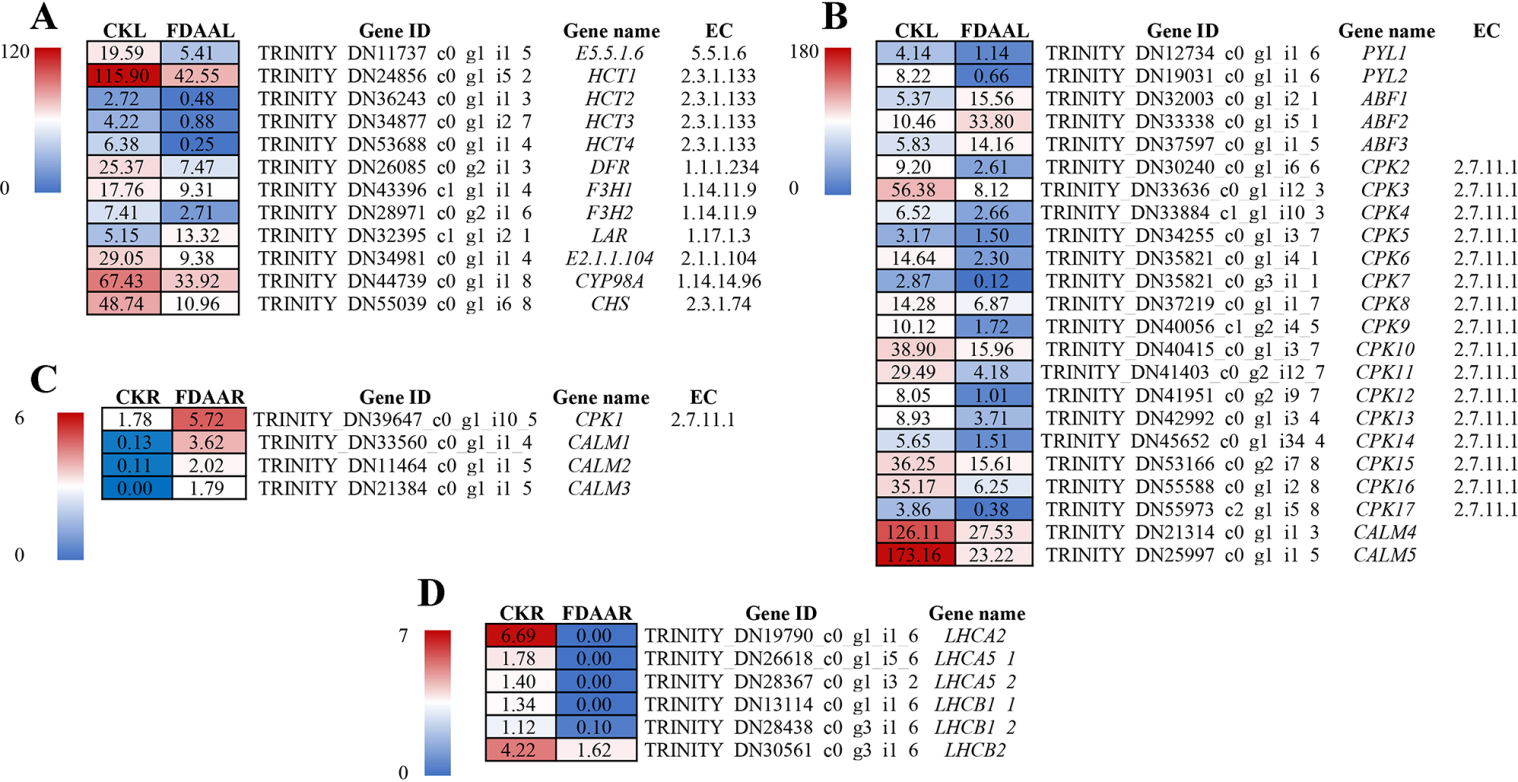
The DEGs related to the flavonoid biosynthesis pathway in FDAAL VS CKL (A) in leaves of *S. tonkinensis* seedlings, the DEGs related to stomatal conductivity in FDAAL VS CKL (B) in leaves of *S. tonkinensis* seedlings, the DEGs related to water absorption in FDAAR VS CKR (C) and the DEGs related to photosynthesis - antenna proteins pathway in FDAAR VS CKR (D) in roots of *S. tonkinensis* seedlings under FDAA treatment. The numbers in blocks represent the average FPKM values of samples in each treatment. These DEGs need to meet the threshold that *p* < 0.05 and |log2FC| > 1. Abbreviations: *CHI*, chalcone isomerase [EC:5.5.1.6]; *HCT*, shikimate O-hydroxycinnamoyl transferase [EC:2.3.1.133]; *DFR*, flavanone 4-reductase [EC:1.1.1.219]; *F3H*, naringenin 3-dioxygenase [EC:1.14.11.9]; *LAR*, leucoanthocyanidin reductase [EC:1.17.1.3]; *E2.1.1.104*, caffeoyl-CoA O-methyltransferase [EC:2.1.1.104]; *CYP98A*, 5-O-(4-coumaroyl)-D-quinate 3’-monooxygenase [EC:1.14.14.96]; *CHS*, chalcone synthase [EC:2.3.1.74]; *PYL*, abscisic acid receptor PYR/PYL family; *ABF*, ABA responsive element binding factor; *CPK*, calcium-dependent protein kinase [EC:2.7.11.1]; *CALM*, calmodulin

After KEGG enrichment analysis, we analyzed 12 DEGs related to “flavonoid biosynthesis” pathway, which was most enriched in the leaves of *S. tonkinensis* under FDAA stress (Fig. 8A). These DEGs were classified to 8 types of genes, including chalcone isomerase (*CHI*), shikimate O-hydroxycinnamoyl transferase (*HCT*), flavanone 4-reductase (*DFR*), naringenin 3-dioxygenase (*F3H*), leucoanthocyanidin reductase (*LAR*), caffeoyl-CoA O-methyltransferase (*E2.1.1.104*), 5-O-(4-coumaroyl)-D-quinate 3’-monooxygenase (*CYP98A*), and chalcone synthase (*CHS*). A total of 11 DEGs in leaves took on down-regulation after FDAA treatments, while only one *LAR* was up-regulated. We also analyzed the DEGs related to water loss and water absorption in leaves and roots of *S. tonkinensis* seedlings under FDAA, respectively. In FDAAL VS CKL, 2 abscisic acid receptor PYR/PYL family (*PYL*) genes were negatively regulated, while 3 ABA responsive element binding factor (*ABF*) genes were positively regulated. A total of 16 *CPK* genes, encoding calcium-dependent protein kinases, were down-regulated. Additionally, 2 calmodulin (*CALM*) genes showed down-regulation (Fig. 8B). In roots, 1 *CPK* gene and 3 *CALM* genes were up-regulated (Fig. 8C). In the photosynthesis - antenna proteins pathway, all DEGs belonging to *LHCA* and *LHCB* took on the downtrend in roots after FDAA treatment (Fig. 8D).

## Discussion

FDAA, a combination of two environmental stresses, including drought damage and waterlogging destruction. To comprehensively investigate how *S. tonkinensis* seedlings respond to FDAA, we analyzed morphological variations, growth conditions, water content, ROS generation, antioxidant enzyme activity, and related key DEGs.

### Impacts of FDAA on growth condition and water content of *S. tonkinensis*

The study was conducted during the growing season for *S. tonkinensis* seedlings. Under normal water management, these seedlings exhibited rapid growth, with a 13.83% increase in H and a 5.41% increase in D. Nevertheless, the growth of *S. tonkinensis* seedlings was significantly inhibited by FDAA and DT treatments. Notably, DT stress had a more pronounced inhibitory effect on the growth of H and D when compared to pre-treatment of DT group, reducing them by 5.25% and 15.74%, respectively. Ünyayar et al. [40] reported that drought stress led to a decline in shoot growth in drought-sensitive *Lycopersicon peruvianum*. Drought stress impaired the shoots growth and roots growth of potato [41]. In addition, the growth and development of cotton was hindered by waterlogging, due to the obstacle to absorbing water and nutrient [42]. FDAA combines the waterlogging and drought stress, which might have superimposed obstruction for the growth of *S. tonkinensis* seedlings. FDAA stress also had a negative effect on rice growth, eventually reducing the rice yield [17]. In terms of the morphological changes, the stems tended to bend, and the leaves exhibited drooping due to water loss. The reduction in water content was particularly concentrated in the leaves exposed to FDAA (Table 2). Combined the results from Tab.S8, the leaf relative water content decreased from 92.97 to 76.50% after two-day-waterlogging. It can be inferred that FDAA represents a form of superimposed damage for *S. tonkinensis* seedlings.

In plants, drought stress is intricately linked to osmotic stress. In general, the rapid elevation of Ca^2+^ levels triggered by these osmotic sensors tends to coincide with alterations in cell membrane tension [43]. According to the transcriptome data, the *CPK* and *CALM* genes were also up-regulated in roots after the drought period of FDAA. The main function of calmodulin (CALM) is to serve as a Ca^2+^ signal transducer [44]. When the concentration of Ca^2+^ increases in plant root systems, it may affect the characteristics of the cell wall, leading to increased rigidity of the cell wall and consequently influencing the root system’s ability to absorb water [45]. More importantly, the root vigor of *S. tonkinensis* seedlings declined by 65.47% after experiencing two-day-waterlogging. Here, the lower root vigor means the higher damage to roots. Thus, we speculated that the water absorption capacity of roots was inhibited due to decreased root vigor, hindering vertical water transport to stems and leaves during the waterlogging period. Combined the results in Table 2, the water content in roots, stems and leaves were decreased due to FDAA, we inferred the water loss from roots resulted from the low ability of water absorption.

In leaves, the down-regulation of *PYL* genes under FDAA would affect the signaling transduction of abscisic acid (ABA) to response in stomatal closure under the drought period of FDAA [46]. During the process of stomatal closure, changes in the concentration of Ca^2+^ can trigger responses in guard cells, leading to changes in intracellular water pressure and consequent closure of the stomata. Specifically, when the concentration of Ca^2+^ increases inside the cell, the cell walls of guard cells become more rigid, causing an increase in intracellular water pressure, which leads to the expansion of guard cells and subsequent closure of the stomata [47, 48]. This process is an adaptive response of plants to drought or other adverse environmental conditions, aimed at reducing water loss through transpiration. However, the genes including *CPK* and *CALM*, which were related to Ca^2+^ regulation, showed down-regulation in our results. In plants, calcium-dependent protein kinases (CPK) play a crucial role in many physiological processes, including stress response and hormone signaling. They are involved in regulating the plant’s response to the external environment. For example, CPK can mediate the plant’s stress response, promoting adaptation to drought stress [48]. Based on the evidence presented, it appeared that the leaves of *S. tonkinensis* seedlings treated with FDAA were unable to effectively regulate stomatal closure, resulting in abundant water loss through continued transpiration from leaves [49].

### Impacts of FDAA on the ROS generation and energy metabolism of *S. tonkinensis*

ROS are molecules characterized by high reactivity that contain oxygen atoms and are generated as by-products of various cellular processes. These ROS play important roles in cell signaling and defense mechanisms but can also be toxic when their levels exceed the cellular capacity to detoxify them [50, 51]. ROS are generated in different cellular sites within plant cells, including peroxisomes, mitochondria, chloroplasts, and the apoplast [51]. Under abiotic stress conditions, accumulation of an excess of ROS occurs as a result of electron leakage from complexes I and III, resulting in the generation of O_2_^−^. This O_2_^−^ is subsequently catalyzed by Mn-SOD and Cu/Zn-SOD to produce H_2_O_2_ [52]. Peroxisomes in various plant species house notable types of SODs, including Cu/Zn-SOD and Mn-SOD, establishing them as crucial locations for H_2_O_2_ production [53, 54]. In non-photosynthetic plant organs, especially in roots, mitochondria are frequently regarded as the primary sites for the generation of ROS. This is because roots rely on mitochondrial respiration for energy production [50]. Zheng et al. [50], He et al. [51], and Da-Silva and do Amarante [52] all provided evidence that waterlogging, a condition where plant roots were submerged in water for an extended period, leading to an elevation in concentrations of ROS in watermelon [55], cucumber [56] and soybean [57]. Drought stress can also cause the imbalance between ROS and antioxidant ability, further generating excessive ROS and leading to leaf senescence [58]. Under drought stress, ROS was accumulated to a high level in *Arabidopsis* [59].

In our study, both FDAA and DT stress conditions led to a noteworthy rise in the concentrations of O_2_^−^ and H_2_O_2_ in the leaves of *S. tonkinensis* seedlings. These ROS are associated with oxidative stress and can have detrimental effects on plant cells [60]. While there was a slight increase in O_2_^−^ and H_2_O_2_ content in the roots as well, these increases were not statistically significant compared to CK, reflecting that the leaves of the plants were more severely affected by oxidative stress in response to FDAA and DT stress. It appeared that FDAA was causing an increase in the generation of ROS, particularly in the leaves of *S. tonkinensis* seedlings, due to the ROS harm could be superimposed. Noctor [49] claimed that photorespiration produced the majority of H_2_O_2_ under drought stress circumstances.

In *A. thaliana* plants, exposure to salt stress leads to the up-regulation of three peroxisome-associated genes: thiolase *(PED1*), peroxin-10 (*PEX10*), and peroxin-1 (*PEX1*) [61]. In *S. tonkinensis* seedlings, peroxin-11 (*PEX11*) gene, which medicates peroxisome proliferation was obviously up-regulated in roots after FDAA treatment [62]. Furthermore, *PEX3* in leaves of *S. tonkinensis* exhibited the highest expression level (18.60), and showed a significant difference compared to other samples. However, the expression level of peroxin-14 (*PEX14*) gene displayed the opposite trend in leaves under DT stress. *MVP17* is able to encode a peroxisomal protein producing ROS, which might regulate the activity of antioxidant enzymes [63, 64]. Under FDAA circumstance, the expression of *MPV17* genes showed the opposite reaction in leaves and roots with up-regulation and down-regulation, respectively. In more specific terms, the down-regulation of the *MPV17* gene in the roots of *S. tonkinensis* seedlings resulted in reduced ROS production (Tab.S7). This suggests that excessive ROS production in the peroxisomes of the roots was not the primary cause of root necrosis under FDAA conditions. It can be inferred that ROS-induced damage primarily accumulated in the leaves of *S. tonkinensis* seedlings during FDAA stress.

In roots of *S. tonkinensis* seedlings under FDAA treatment, the majority of top 20 KEGG enrichment pathways were found to be associated with energy metabolism, including pathways related to photosynthesis and glycolysis. Interestingly, it was observed that most DEGs involved in these pathways were significantly down-regulated (Tab.S7). For example, all DEGs encoding LHCA (light-harvesting complex I chlorophyll a/b binding protein) and LHCB (light-harvesting complex II chlorophyll a/b binding protein) maintained a downtrend following FDAA treatment. This phenomenon not only suggests a reduced need for exogenous carbon in *S. tonkinensis* roots but also reflects an enhancement in FDAA tolerance [65]0.4.3 Impacts of FDAA on antioxidant system enzymes and flavonoid biosynthesis of *S. tonkinensis*.

The antioxidant system preserves plants from oxidative damage under diverse environmental stresses [51]. Under waterlogging and drought stress, the antioxidant system helps to scavenge ROS that accumulate in plant tissues due to limited oxygen availability and water deficit, respectively [66, 67]. The antioxidant enzymes are up-regulated to detoxify ROS and maintain cellular redox homeostasis [68]. SOD and CAT are two important enzymes involved in the defense mechanisms of plants against drought and flooding stress [69, 70]. SOD plays a crucial role in scavenging ROS generated during drought stress. It converts superoxide radicals into H_2_O_2_, which is then detoxified by CAT [71]. In summary, SOD plays a crucial role in scavenging ROS, while CAT aids in the detoxification of H_2_O_2_, thereby maintaining the balance between ROS production and scavenging. In our study, it was observed the elevated activities of SOD and CAT in two organs of *S. tonkinensis* seedlings under FDAA and DT stress conditions. These findings were consistent with previous research on strawberries, where an increase in SOD and CAT activities in strawberry leaves exposed to drought stress [72]. Similar responses were also observed in *Bupleurum chinense* under drought stress [73]. In a study conducted on potato genotypes under water deficit conditions, it was observed that the activity of SOD, including Fe-SOD isoforms, resulted in an enhancement of water use efficiency (WUE) [74]. Bansal and Srivastava [65] also discovered that waterlogging triggered an increase in CAT and SOD activities in *Cajanus cajan*. Furthermore, the cultivar with higher waterlogging-resistance or drought resistance exhibit higher antioxidant enzyme activity [75–77]. Besides, a drought-resistant variety exhibits a more effective mechanism for scavenging ROS, as evidenced by a significant boost in the activity of the antioxidant enzyme SOD [76]. Compared to FDAA stress, SOD activities in leaves and roots were higher under DT condition. However, CAT activities presented the opposite performance. Combined the ROS condition, it was inferred that SOD activity was inhibited by FDAA stress to scavenge less O_2_^−^ in leaves of *S. tonkinensis* seedlings, which was displayed in Fig. 2A and Fig.S1. Additionally, the higher CAT activity was accord with lower H_2_O_2_ content (Fig. 1B and Fig.S1), due to the essential role of CAT in breaking down H_2_O_2_ into water and oxygen [71].

Multiple transcriptomic investigations have demonstrated a robust correlation between peroxisomal H_2_O_2_ and oxidative stress. This suggests that the balance of redox homeostasis, which is connected to the NAD and NADP systems, could potentially regulate this interaction [78–80]. Three genes in *Arabidopsis thaliana*, encoding catalase have been discovered. The expression of catalase-2 (*CAT2*) is linked to the photorespiration pathway, while catalase-1 (*CAT1*) expression is connected to fatty acid β-oxidation. catalase-3 (*CAT3*), on the other hand, is associated with senescence processes [81, 82]. In the study, the expression levels of *CAT* were elevated in the leaves and roots of *S. tonkinensis* seedlings under both FDAA and DT stresses, except for the roots under FDAA condition, which was not obvious DEGs. In rice chloroplasts, the overexpression of a pea manganese *SOD* gene (*MnSOD*) controlled by an oxidative stress-inducible promoter *SWPA2* has been found to enhance the drought tolerance of transgenic rice [83]. In general, Cu/Zn family superoxide dismutase (*SOD1*) and Fe/Mn family superoxide dismutase (*SOD2*) genes in *S. tonkinensis* seedlings were up-regulated to combat abiotic stresses. Nevertheless, *SOD2* presented obviously down-regulated in the leaves under DT condition. In Tab.S9, it was displayed that the significant correlation between SOD activity and the gene expression of *SOD2* in both leaves and roots.

In response to oxidative damage, plants activate the production of antioxidant enzymes. However, when faced with severe environmental stress, plants may struggle to produce enough antioxidants to counter the oxidation, resulting in elevated levels of reactive ROS within cells. In such challenging conditions, flavonoids play a crucial role in mitigating excessive ROS production and repairing associated damage [84]. Flavonoids, a diverse class of secondary metabolites, exhibit robust antioxidant properties that support plants in coping with various environmental stresses [85]. In one process of flavonoid biosynthesis, chalcone synthase and chalcone isomerase are enzymes responsible for a two-step condensation process, resulting in the production of naringenin. In our results, *CHS* and *CHI*, which respectively regulated the biosynthesis of these enzymes, were down-regulated. Subsequently, *F3H* encodes naringenin 3-dioxygenase to generate dihydrokae mpferol. After *DFR* encodes flavanone 4-reductase to catalyze dihydrokaempferol to leucopelargonidin, anthocyanidin synthase converts leucopelargonidin to pelargonidin, which is then sent to anthocyanin biosynthesis. Moreover, the up-regulated *LAR* encodes leucoanthocyanidin reductase to turn leucopelargonidin to afzelechin, hindering the biosynthesis of pelargonidin [86]. Abundant evidence has proven that the genes involved in “flavonoid biosynthesis” attempt to improve their expression level to counter abiotic stresses [85, 87]. For instance, *Reaumuria soongorica*, a desert plant, could combat drought stress through positive regulation of *RsF3H* gene [88]. Conversely, a total of 11 DEGs in the flavonoid biosynthesis pathway were significantly down-regulated, indicating that these genes in the leaves of *S. tonkinensis* seedlings did not effectively scavenge ROS.

**Fig. 9:**
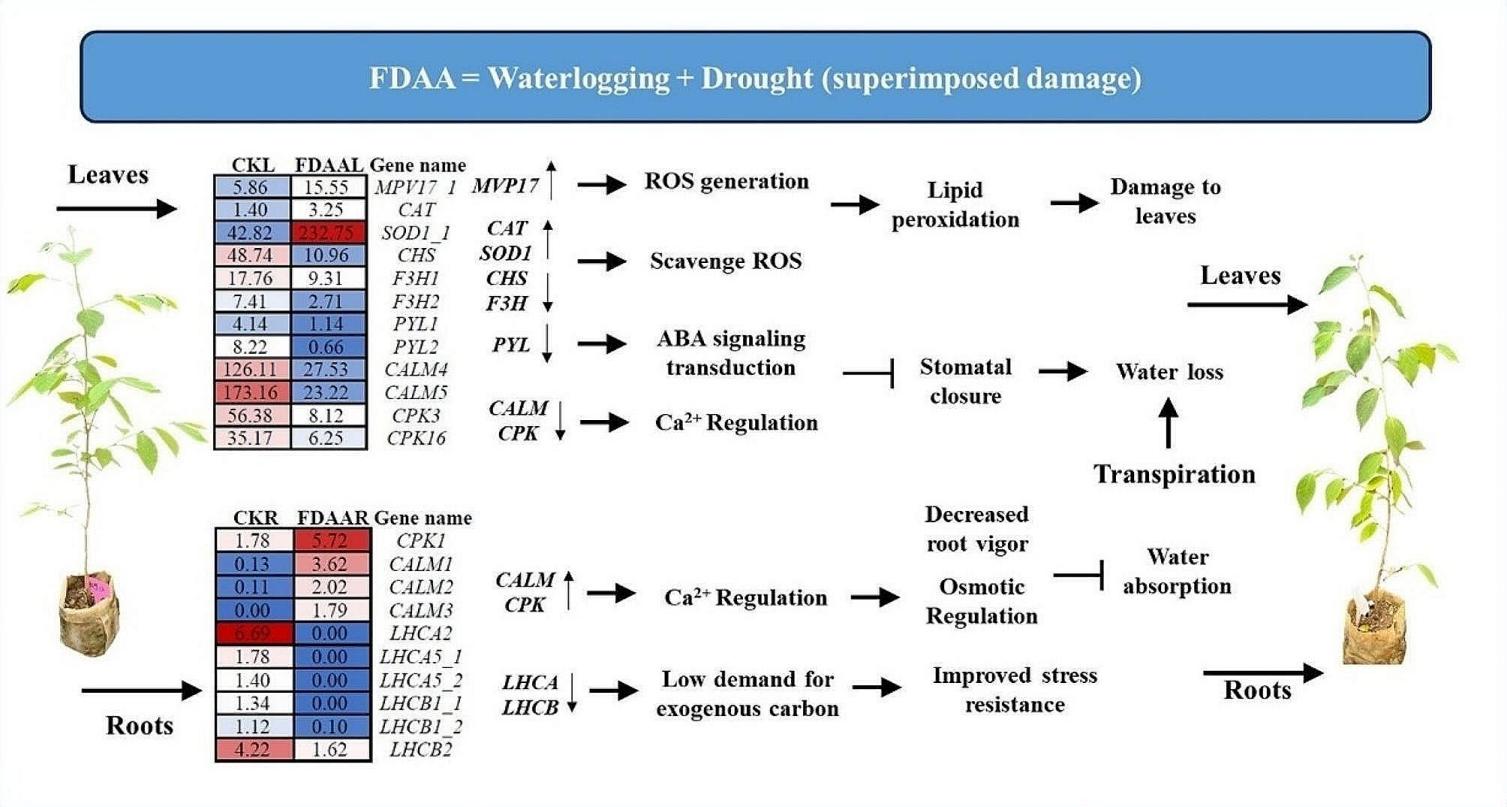
A network regulation model of *S. tonkinensis* seedlings under FDAA. The numbers in left and right blocks represent the average FPKM values of samples in CK and FDAA, respectively. These DEGs need to meet the threshold that *p* < 0.05 and |log2FC| > 1

Overall, FDAA represents a compounded impact of waterlogging and drought. Under FDAA, the up-regulation of the *MVP17* gene in leaves triggered ROS accumulation, consequently causing lipid peroxidation and damage to leaves of *S. tonkinensis* seedlings. The down-regulation of *CHS* and *F3H* did not play an essential role in scavenging ROS. Additionally, the down-regulation of *PYL*, *CALM*, and *CPK* presented impediments to stomatal closure. This damage to the leaves, coupled with inhibited stomatal closure and sustained transpiration, exacerbated water loss from the leaves. In the roots, the upregulation of *CALM* and *CPK*, coupled with decreased root vigor, resulted in reduced water absorption by the roots. Besides, the DEGs in the photosynthesis - antenna proteins including *LHCA* and *LHCB* decreased their gene expression, indicating low demand for exogenous carbon (Fig. 9).

## Conclusion

The entire study progressively reveals the responses of *S. tonkinensis* seedlings to rapid shifts from waterlogging to drought stress, examining their responses at the morphological, physiological, and molecular levels. Additionally, it compares the effects of DT stress and FDAA stress on the seedlings. Furthermore, we observed that the accumulation of ROS induced by waterlogging and drought stress during this rapid transition is additive, with the primary damage occurring predominantly in the leaf tissues. The ultimate cause of plant mortality may be attributed to water loss during the waterlogging phase, diminished root water uptake capacity, and continued water loss during the subsequent drought period.

### Electronic supplementary material

Below is the link to the electronic supplementary material.

Supplementary Material 1

Supplementary Material 2

Supplementary Material 3

Supplementary Material 4

Supplementary Material 5

## Data Availability

Sequence data that support the findings of this study have been deposited in China National Center for Bioinformation (CNCB) with the primary accession code CRA012350.

## Abbreviations

ABA: Abscisic acid
*ABF*: ABA responsive element binding factor
ANOVA: Analysis of variance
BLAST: Basic local alignment search tool
*CALM*: Calmodulin
CAT: Catalase
*CAT1*: Catalase-1
*CAT2*: Catalase-2
*CAT3*: Catalase-3
*CHI*: Chalcone isomerase
*CHS*: Chalcone synthase
*CPK*: Calcium-dependent protein kinase
CK: Control groups
CKL: Leaf sample in control groups
CKR: Root sample in control groups
D: Shoot diameter
*CYP98A*: 5-O-(4-coumaroyl)-D-quinate 3’-monooxygenase
DAB: 3,3’-diaminobenzidine
DEGs: Differentially expressed genes
*DFR*: Flavanone 4-reductase
DT: Drought stress
DTL: Leaf sample under drought stress
DTR: Root sample under drought stress
*E2.1.1.104*: Caffeoyl-CoA O-methyltransferase
eggnog: Evolutionary genealogy of genes: non-supervised orthologous groups
*F3H*: Naringenin 3-dioxygenase
FDAA: Flood-drought abrupt alternation
FDAAL: Leaf sample under flood-drought abrupt alternation
FDAAR: Root sample under flood-drought abrupt alternation
FPKM: Fragments per kilobase per million mapped reads
GO: Gene ontology
H: Seedling height
H_2_O_2_: Hydrogen peroxide
*HCT*: Shikimate O-hydroxycinnamoyl transferase
KEGG: Kyoto encyclopedia of genes and genomes
KOG: Clusters of orthologous groups
*LAR*: Leucoanthocyanidin reductase
MDA: Malondialdehyde
*MnSOD*: Manganese *SOD* gene
*MPV17*: Protein Mpv17
NB: Negative binomial
NBT: Nitrozolium blue tetrachloride
O_2_^−^: Superoxide anion
PBS: Phosphate buffer solution
*PED1*: Thiolase
*PEX1*: Peroxin-1
*PEX10*: Peroxin-10
*PEX11*: Peroxin-11
*PEX14*: Peroxin-14
*PMP34*: Peroxisomal adenine nucleotide transporter
*PYL*: Abscisic acid receptor PYR/PYL family
ROS: Reactive oxygen species
SOD: Superoxide dismutase
*SOD1*: Cu/Zn family superoxide dismutase
*SOD2*: Fe/Mn family superoxide dismutase
SwissProt: Swiss-Prot protein
TCA: Trichloroacetic acid
