# Supplementary figures and images for: Transcriptome analysis of antioxidant system response in Styrax tonkinensis seedlings under flood-drought abrupt alternation
Source: BMC Plant Biol. 2024 May 17;24:413. doi: 10.1186/s12870-024-05130-4 (PMC11100094; doi:10.1186/s12870-024-05130-4)

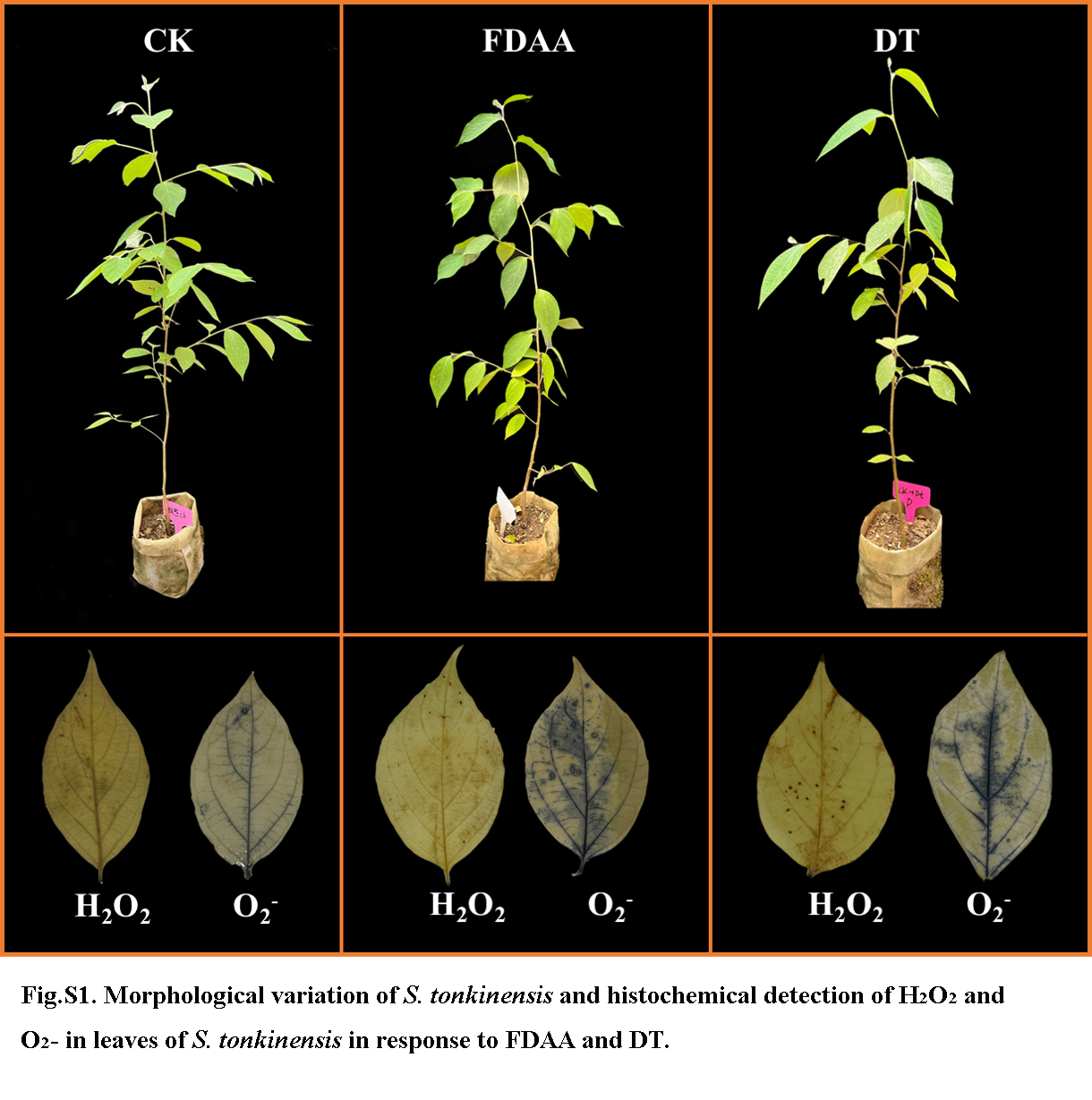

Supplement: Supplementary file 1 — Supplementary Material 1 [file 12870_2024_5130_MOESM1_ESM.jpg]

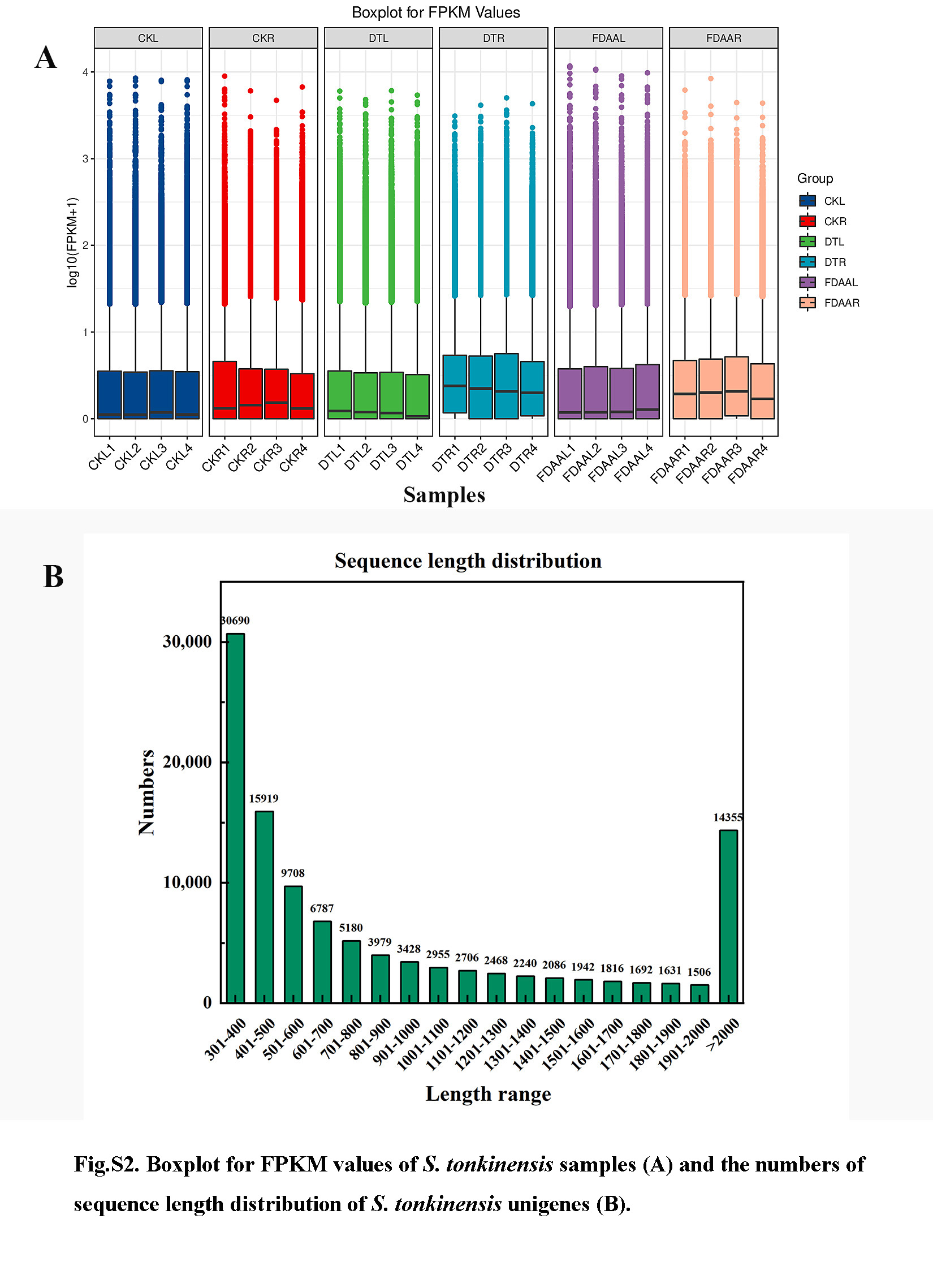

Supplement: Supplementary file 2 — Supplementary Material 2 [file 12870_2024_5130_MOESM2_ESM.jpg]

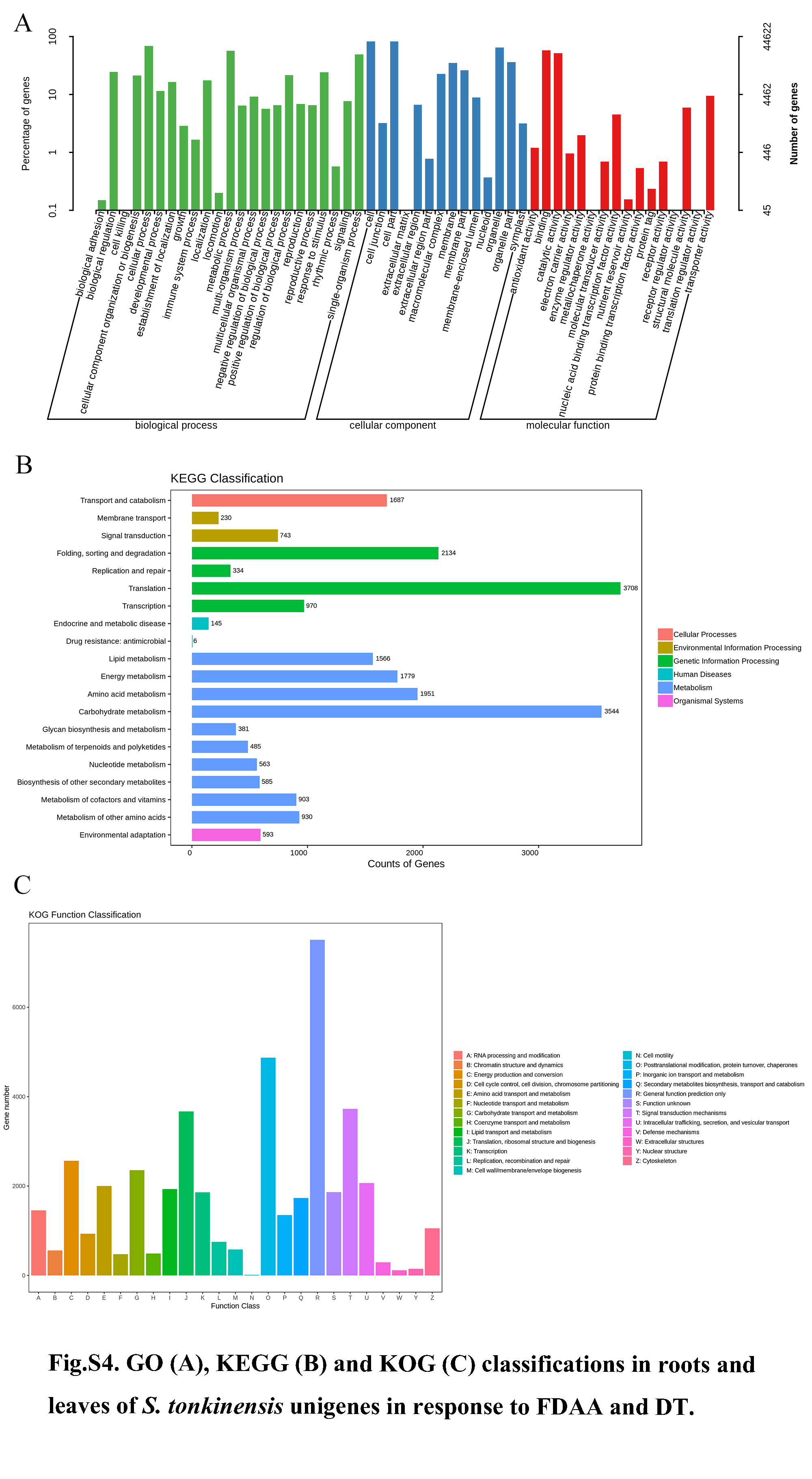

Supplement: Supplementary file 3 — Supplementary Material 3 [file 12870_2024_5130_MOESM3_ESM.jpg]

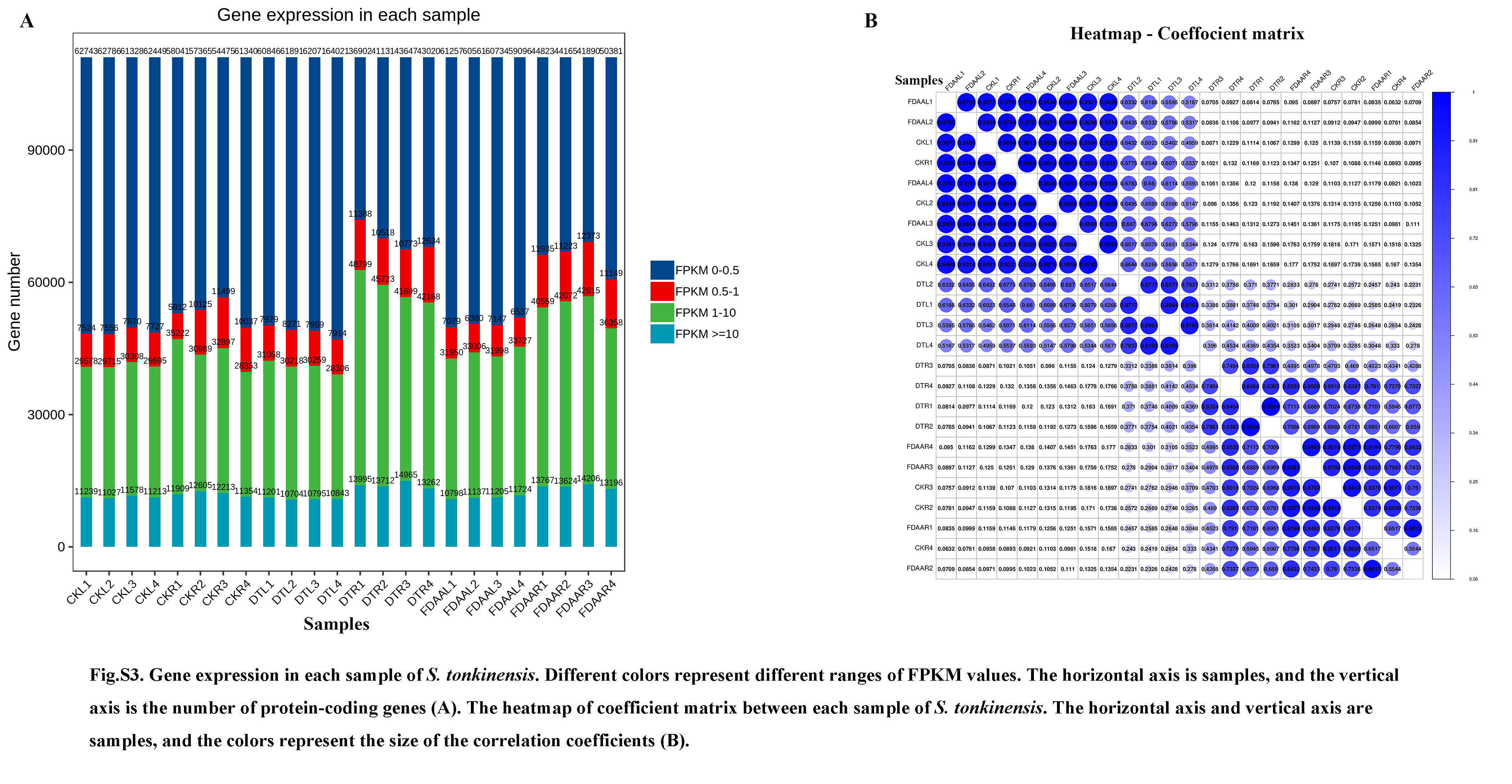

Supplement: Supplementary file 4 — Supplementary Material 4 [file 12870_2024_5130_MOESM4_ESM.jpg]
